# Association between Pre-Treatment Biological Indicators and Compliance to Neoadjuvant/Perioperative Chemotherapy in Operable Gastric Cancer

**DOI:** 10.3390/nu15163604

**Published:** 2023-08-17

**Authors:** Manlio Monti, Andrea Prochowski Iamurri, David Bianchini, Chiara Gallio, Luca Esposito, Daniela Montanari, Silvia Ruscelli, Chiara Molinari, Flavia Foca, Alessandro Passardi, Giovanni Vittimberga, Paolo Morgagni, Giovanni Luca Frassineti

**Affiliations:** 1 Department of Medical Oncology, IRCCS Istituto Romagnolo per lo Studio dei Tumori (IRST) “Dino Amadori”, 47014 Meldola, Italy; 2 Radiology Unit, IRCCS Istituto Romagnolo per lo Studio dei Tumori (IRST) “Dino Amadori”, 47014 Meldola, Italy; 3 Medical Physics Unit, IRCCS Istituto Romagnolo per lo Studio dei Tumori (IRST) “Dino Amadori”, 47014 Meldola, Italy; 4 Biosciences Laboratory, IRCCS Istituto Romagnolo per lo Studio dei Tumori (IRST) “Dino Amadori”, 47014 Meldola, Italy; 5 Unit of Biostatistics and Clinical Trials, IRCCS Istituto Romagnolo per lo Studio dei Tumori (IRST) “Dino Amadori”, 47014 Meldola, Italy; 6 General and Oncologic Surgery, “Morgagni-Pierantoni” Hospital, 47121 Forlì, Italy

**Keywords:** locally advanced gastric cancer, neoadjuvant, perioperative chemotherapy, compliance, pre-treatment indicators

## Abstract

Background and aims: Perioperative treatment is currently the gold standard approach in Europe for locally advanced gastric cancer (GC). Unfortunately, the phenomenon of patients dropping out of treatment has been frequently observed. The primary aims of this study were to verify if routine blood parameters, inflammatory response markers, sarcopenia, and the depletion of adipose tissues were associated with compliance to neoadjuvant/perioperative chemotherapy. Methods and study design: Blood samples were considered before the first and second cycles of chemotherapy. Sarcopenia and adipose indices were calculated with a CT scan before starting chemotherapy and before surgery. Odds ratios (OR) from univariable and multivariable models were calculated with a 95% confidence interval (95% CI). Results: A total of 84 patients with locally advanced GC were identified between September 2010 and January 2021. Forty-four patients (52.4%) did not complete the treatment according to the number of cycles planned/performed. Eight patients (9.5%) decided to suspend chemotherapy, seven patients (8.3%) discontinued because of clinical decisions, fourteen patients (16.7%) discontinued because of toxicity and fifteen patients (17.9%) discontinued for miscellaneous causes. Seventy-nine (94%) out of eighty-four patients underwent gastrectomy, with four patients having surgical complications, which led to a suspension of treatment. Sarcopenia was present in 38 patients (50.7%) before chemotherapy began, while it was present in 47 patients (60%) at the CT scan before the gastrectomy. At the univariable analysis, patients with basal platelet to lymphocyte ratio (PLR) ≥ 152 (*p* = 0.017) and a second value of PLR ≥ 131 (*p* = 0.007) were more frequently associated with an interruption of chemotherapy. Patients with increased PLR (*p* = 0.034) compared to the cut-off were associated with an interruption of chemotherapy, while patients with increased monocytes between the first and second cycles were associated with a lower risk of treatment interruption (*p* = 0.006); patients who underwent 5-fluorouracil plus cisplatin or oxaliplatin had a higher risk of interruption (*p* = 0.016) compared to patients who underwent a 5-fluorouracil plus leucovorin, oxaliplatin and docetaxel (FLOT) regimen. The multivariable analysis showed a higher risk of interruption for patients who had higher values of PLR compared to the identified cut-off both at pretreatment and second-cycle evaluation (OR: 5.03; 95% CI: 1.34–18.89; *p* = 0.017) as well as for patients who had a lower PLR than the identified cut-off at pretreatment evaluation and had a higher PLR value than the cut-off at the second cycle (OR: 4.64; 95% CI: 1.02–21.02; *p* = 0.047). Becker regression was neither affected by a decrease of sarcopenia ≥ 5% (*p* = 0.867) nor by incomplete compliance with chemotherapy (*p* = 0.281). Conclusions: Changes in PLR values which tend to increase more than the cut-off seem to be an immediate indicator of incomplete compliance with neoadjuvant/perioperative treatment. Fat loss and sarcopenia do not appear to be related to compliance. More information is needed to reduce the causes of interruption.

## 1. Introduction

Globally, gastric cancer (GC) is the third leading cause of cancer death [1], despite its declining incidence. Preoperative treatment offers a promising approach for locally advanced GC. Based on three studies [2,3,4], the European Society for Medical Oncology guidelines [5] indicate perioperative treatment for almost all GC patients in Western countries. The studies [2,3,4] showed that perioperative chemotherapy was completed by approximately 34–50% of GC patients. Therefore, less than half of the patients completed perioperative chemotherapy and only a small number of patients (2–16%) benefited from complete pathological remission [6]. Hence, there is a need to identify the host factors and/or tumor factors and/or kinds of treatment that are associated with treatment compliance and therapeutic activity/efficacy. Myelosuppression is one of the most common side effects of chemotherapy, and myelosuppression is a frequent cause of chemotherapy discontinuation. It may be useful to assess the blood values which express bone marrow, renal and hepatic function and assess the inflammatory response markers such as neutrophils-to-lymphocytes ratio (NLR), derivative NLR (d-NLR) calculated as neutrophils/absolute white blood cells–neutrophils ratio, PLR and the monocytes-to-lymphocytes ratio (MLR), which have been widely proposed as prognostic factors for several malignancies [7,8,9,10,11,12]. The inflammatory response markers represent the patient’s state of well-being.

Sarcopenia is associated with changes in the inflammatory state [13] and, therefore, might affect patients’ ability to complete chemotherapy and perhaps how they respond to the chemotherapy itself. Hence, we think it is necessary to investigate whether routine blood parameters and inflammatory ratio can predict compliance with preoperative chemotherapy in GC as well as sarcopenia and or loss of adipose tissue.

## 2. Materials and Methods

### 2.1. Patients’ Characteristics

This retrospective study assessed the electronic data of 26 patients enrolled in the IRST151.01 GASTRODOC study [14] from September 2010 to September 2017 in the Forlì-Cesena catchment area and a further 58 patients treated from September 2010 to January 2021 in the same area.

We reviewed medical records, identifying all consecutive patients with a histologic diagnosis of GC treated with neoadjuvant/perioperative chemotherapy at the IRCSS Istituto Romagnolo per lo Studio dei Tumori (IRST) “Dino Amadori”. An electronic CRF collected demographic data (age, sex, height, weight, Eastern Cooperative Oncology Group (ECOG) performance status (PS), body-mass index (BMI)), clinical data (histopathology, grading, tumor location, cTNM, pathological response), information on treatment such as surgery or chemotherapy, relapse and follow-up data.

The staging system included a chest, abdomen and pelvis computed tomography (CT) scan. Gastroscopy and CT scans of the thorax and abdomen had to be carried out no later than three weeks before chemotherapy began. The CT scan was repeated before the gastrectomy. The 7th edition UICC TNM was used for the staging classification [15]. Tumors located proximally were classified according to Siewert and Stein [16]. Siewert type I and II tumors were excluded from the analysis. 

Inclusion criteria included patients of either gender with locally advanced (≥T3 or bulky N+) GC, age ≥ 18 years, ECOG performance status 0–1 at study entry and any type of neoadjuvant or perioperative chemotherapy. The definition of “bulky lymph node metastases” is in line with the indications of Yoshikawa et al. [17]: “at least one node of ≥3 cm in diameter or at least three consecutive nodes of ≥1.5 cm diameter in first or second level lymph node stations”.

Exclusion criteria included early GC (if N0), T2 if N0, linitis plastic, positive peritoneal cytology, distant metastases, peritoneal involvement, concurrent chronic systemic immunotherapy, corticosteroid therapy four weeks before study entry and any concurrent malignancy. A multidisciplinary team evaluated each case.

### 2.2. Endpoints

The primary aim was to verify if the absolute values or variations in white blood cells, neutrophils, lymphocytes, monocytes, platelets, hemoglobin (Hgb), inflammatory indices (NLR, dNLR, PLR, MLR, lymphocytes-to-white blood cells ratio (LWR), lymphocytes-to-monocytes ratio (LMR), platelets × neutrophils/lymphocytes (SII), systemic inflammation response index (SIRI) based on neutrophils × monocytes/lymphocytes, estimated glomerular filtration rate (eGFR), total bilirubin (TBIL), sarcopenia, depletion of adipose tissues, type of chemotherapy were associated with compliance to neoadjuvant/perioperative chemotherapy.

The secondary aims were to verify (1) if parameters analyzed for primary aim were associated with pathological response; (2) the frequency of sarcopenia before starting chemotherapy and before surgery; (3) if survival depended on compliance.

We defined total compliance as the patient undergoing the number of treatment cycles estimated before starting chemotherapy, including intervention and post-surgery chemotherapy if it had been planned at the first visit. The tumor regression grade was assessed using the Becker regression criteria [18], which estimate the percentage of vital tumor cells concerning the macroscopically identified tumor bed and includes the following categories: 1a (pathological complete remission), 1b (subtotal regression: <10% residual tumor cells), 2 (partial regression: 10–50% residual tumor cells) and 3 (minor or no regression: >50% residual tumor cells).

### 2.3. Anthropometric Measurements, Body Composition Measurements and Sarcopenia Assessment

Weight and height were recorded following standard methods. Weight was measured using a medical balance beam scale and height was measured using a stadiometer. The body mass index (BMI) was calculated (weight (kg)/height (m^2^)). A BMI of <20 is considered underweight, a BMI of 20–24 is normal, a BMI of 25–29 is overweight and a BMI > 30 is obese [19].

Weight was collected at baseline and before the first cycle of chemotherapy. The BMI was collected before the first cycle of neoadjuvant chemotherapy.

Patients underwent contrast-enhanced chest and abdominal/pelvic CT scans as part of routine staging workups. The imaging material of patients who were referred from other centers was obtained where possible.

The CT scans shared similar acquisition parameters (tube voltage between 100 and 120 kVp, automatic tube current, soft tissue reconstruction algorithm, a 512 × 512 matrix; and a slice thickness of 5 mm). From the CT scans, a single unenhanced CT image was processed at L3 body level with both transverse processes depictable using ABACS software (version 2.0, Voronoi Health Analytics, Vancouver, BC, Canada), which automatically segmented and measured the cross-sectional surfaces of skeletal muscles (*TMA*), subcutaneous adipose tissue (SAT), intramuscular fat tissue (IMAT) and visceral adipose tissue (VAT) (https://doi.org/10.48550/arXiv.2106.00652, accessed on 17 December 2020). A radiologist blinded to outcomes and with previous experience in this area reviewed, corrected and validated the segmentations and measurements. Patient height was used to calculate the skeletal muscle index (*SMI*) as follows [20]:

$$SMI\;\frac{cm^{2}}{m^{2}}=\frac{TMA\;\left(cm^{2}\right)}{height{\;}^{2}\;\left(m^{2}\right)}$$

Sarcopenia was defined as an *SMI* lower than 41 cm^2^/m^2^ in women, lower than 43 cm^2^/m^2^ in men with a BMI < 25 kg/m^2^ and lower than 53 cm^2^/m^2^ in men with a BMI ≥ 25 kg/m^2^, as described by Martin et al. [21]. Sarcopenic obesity was defined as sarcopenia in patients with a BMI ≥ 25 kg/m^2^.

The subcutaneous adipose tissue index (SATI), visceral adipose tissue index (VATI) and intermuscular adipose tissue index (IMATI) were calculated and correspond, respectively, to the surfaces of SAT, VAT or IMAT divided for height^2^.

The body composition data were evaluated at the time of cancer diagnosis using CT scans (before the first course of chemotherapy) and after terminating neoadjuvant chemotherapy (before gastrectomy, if performed).

### 2.4. Peripheral Venous Blood Sample

Peripheral venous blood samples were taken each morning, 1–2 days before each cycle of chemotherapy. The samples were obtained from patients with an empty stomach and were collected into an ethylene-diamine-tetra-acetic acid (EDTA) tube for measuring blood-routine parameters, including neutrophils, lymphocytes, monocytes, white blood cells, platelets and Hgb. The blood examination also assessed creatinine, eGFR and TBIL values. The time from blood drawing to clinical analysis was less than four hours. From the single blood-routine parameters, we also calculated NLR, dNLR, MLR, LMR, LWR, PLR, SII and SIRI. The blood samples were assessed and reported at baseline (before the first course of chemotherapy) and before the second course of chemotherapy.

Evaluating creatinine and eGFR values is useful to determine renal function [22]. Our evaluation of an eGFR cut-off <60 mL/min/1.73 m^2^ corresponded to chronic kidney disease of grade 2 [23].

A state of anemia was considered as Hgb < 11 g/dL considering a meta-analysis in preoperative GC patients [24], while we considered a cut-off TBIL of 0.32 mg/dL, which corresponded to about 5.3 µmol/L [25].

### 2.5. Treatment Protocols

The GASTRODOC trial considered four courses of neoadjuvant docetaxel/oxaliplatin/capecitabine (DOC) chemotherapy in the neoadjuvant arm and two preoperative and two postoperative DOC treatments in the perioperative arm. DOC chemotherapy consisted of docetaxel 35 mg/m^2^ on days 1 and 8 in a one-hour infusion, oxaliplatin 80 mg/m^2^ on day 1 in a two-hour infusion and capecitabine 750 mg/m^2^ twice daily for two weeks. Each cycle was repeated every three weeks.

Chemotherapy with cisplatin/5-fluorouracil (CF) consisted of three preoperative cycles of intravenous cisplatin 100 mg/m^2^ on day 1 and a continuous intravenous infusion of 5-fluorouracil 800 mg/m^2^/day for five consecutive days (days 1 to 5) every 28 days and three postoperative cycles of the same regimen. 

FOLFOX-4 regimen consisted of six preoperative and six postoperative cycles of oxaliplatin 85 mg/m^2^ as a 2 h infusion on day 1 and a 2 h infusion of calcium levofolinate 200 mg/m^2^/day followed by a 5-fluorouracil bolus 400 mg/m^2^ day and 22 h infusion of 5-fluorouracil 600 mg/m^2^/day for two consecutive days every two weeks. 

The FOLFOX-6 regimen consisted of six preoperative and six postoperative cycles oxaliplatin 85 mg/m^2^ day 1, calcium levofolinate 200 mg/m^2^ day 1, 5-fluorouracil bolus 400 mg/m^2^ day 1, 5-fluorouracil 2400 mg/m^2^ day 1 in 48 h. 

FLOT considered four pre-operative and four post-operative 2-week cycles of 50 mg/m^2^ docetaxel, 85 mg/m^2^ oxaliplatin, 200 mg/m^2^ leucovorin and 2600 mg/m^2^ 5-fluorouracil as a 24 h infusion on day 1.

On day 1 of every 3-week cycle, a bolus of epirubicin at a dose of 50 mg/m^2^, cisplatin at a dose of 60 mg/m^2^ in the ECF and ECX groups and oxaliplatin at a dose of 130 mg/m^2^ were administered intravenously during 2 h in the EOX group. 5-fluorouracil at a daily dose of 200 mg/m^2^ and capecitabine at a twice-daily dose of 625 mg/m^2^ were given throughout treatment in the appropriate groups.

### 2.6. Ethical Approval

The study was approved by the IRST and Wide Area Romagna Ethics Committee (Protocol Code: IRST151.03 IRST—Identifier Code: L2P2176); it also followed the Good Clinical Practice guidelines, as well as the principles laid down at the 18th World Medical Assembly (Helsinki, Finland, 1964), 59th World Medical General Assembly (Seoul, South Korea, October 2008), Directive 2001/20/EC of the European Parliament and other relevant local legislation. Written informed consent was obtained from all patients before the research began. For the patients enrolled outside IRST151.01 GASTRODOC study, the principal investigator signed a Substitutive Informed Consent Declaration Form, declaring that subjects were not expected to sign a specific informed consent if they were unreachable. 

### 2.7. Follow-Up

Follow-up included laboratory exams and clinical visits scheduled every three months for the first, second and third year, until disease progression. After the fourth year, a follow-up was scheduled every six months. Radiological assessment (abdomen CT and chest CT or X-ray) was performed every six months for a maximum of five years, or until disease progression. Gastroscopy was performed one year after randomization, every year thereafter in patients undergoing subtotal gastrectomy and every two years for total gastrectomy for a maximum of five years.

### 2.8. Statistical Analysis

The data were summarized according to median and minimum–maximum for continuous variables and according to frequency and percentage for categorical variables. Associations among potential prognostic factors and treatment interruption were assessed using Chi-square or Fisher exact tests as appropriate. Receiver operating characteristics (ROC) analysis was performed and the area under the curve (AUC) was determined to evaluate continuous values of the inflammatory index before the first and second treatment of chemotherapy as predictors for compliance with treatment. The Youden’s index was calculated and used to determine the cut-offs that gave the best combination of sensitivity and specificity. Based on values reported from indexes at pre-therapy and second cycle, patients were divided into four groups: patients who had values lower than the ROC cut-off at both times, patients who had an increase in the index’s value between baseline and the second cycle, patients who had a decrease and patients who had a higher value than the ROC cut-off both at baseline and the second cycle.

Univariable and multivariable logistic regression models were carried out to assess the independent prognostic role for the interruption of treatment, and odds ratio (OR) with 95% confidence interval were calculated (95%CI), considered as all patients who did not exhibit compliance. The Akaike Information Criteria (AIC) and the Bayesian Information Criteria (BIC) were used for multivariable model comparison considering the fit and complexity of the models. Overall survival (OS) was defined as the length of time from the first day of treatment to death from any cause. Similarly, progression-free survival (PFS) was computed from the initiation of treatment to the date of disease progression or death from any cause, whichever came first. PFS and OS were reported as median values with a 95% confidence interval (95% CI). Survival curves were estimated using the product-limit method of the Kaplan–Meier method (two-sided 95% CIs) and compared with the log-rank test. Cox regression model was carried out to estimate hazard ratio (HR) for PFS and OS. Each analysis evaluated patients who had data available, such as CT scans, Becker regression or information on the progressive disease or alive status. All *p*-values, based on two-sided testing and a *p*-value < 0.05, were considered statistically significant. All statistical analyses were performed using Stata/SE version 15.1 for Windows (StataCorp LP, College Station, TX, USA).

## 3. Results

### 3.1. Demographic Characteristics

Between September 2010 and January 2021, 89 patients were considered, but only 84 patients were eligible for the final analysis, while 5 patients were not considered because of missing data. There were 55 (65.5%) males and 29 (34.5%) females with a median age of 67.2 years (range 22.8–80.3). The median follow-up was 44.8 months (range: 2.9–119.0), while the median time from the start of chemotherapy to surgery was 3.2 months (range: 0.2–5.6). Perioperative chemotherapy was proposed to 59 patients (70.2%) while neoadjuvant chemotherapy was proposed to the remaining 25 patients (29.8%). 

### 3.2. Characteristics of Drop Out

**Figure 1:**
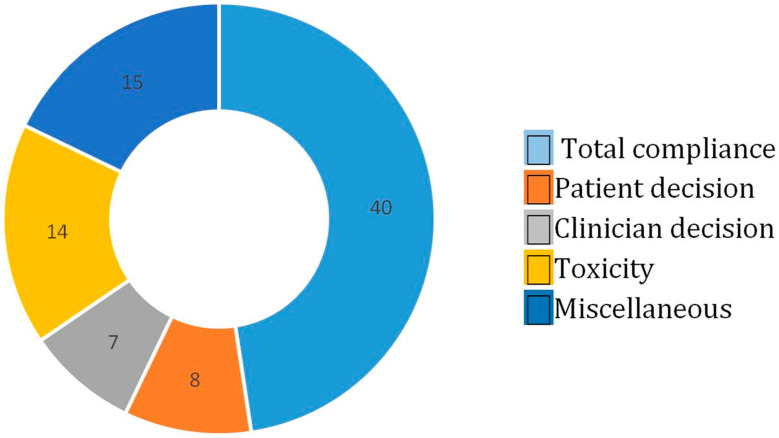
Total compliance vs. causes of non-adhesion.

**Figure 2:**
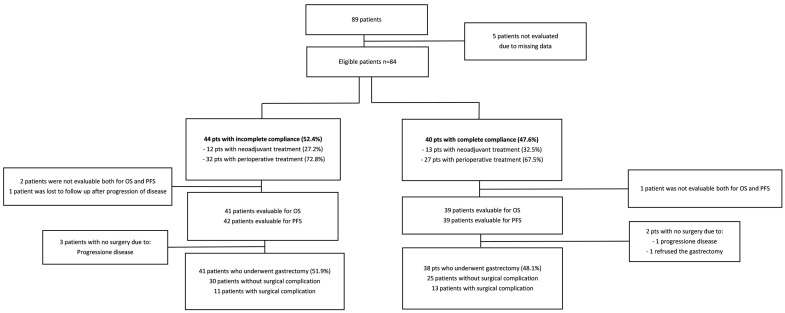
Diagram with the distribution of patients considering compliance, neoadjuvant/perioperative chemotherapy, surgery, and surgical complications.

Forty-four patients (52.4%) did not have total compliance with the treatment according to the number of cycles planned/performed. Eight patients (9.5%) decided to suspend chemotherapy, seven patients (8.3%) discontinued because of clinical decision, fourteen patients (16.7%) discontinued because of toxicity and fifteen patients (17.9%) discontinued for miscellaneous reasons (four progression diseases, two gastric perforations, four surgical complications, two thromboembolism/brain stroke, two worsening of PS and one weight loss) (Figure 1). Twelve (27.2%) out of the fourty-four patients had neoadjuvant chemotherapy, while the remaining thirty-two patients (72.8%) had a perioperative treatment, respectively. Among 40 patients (47.6%) that maintained total compliance with the therapeutic program, 13 patients (32.5%) had neoadjuvant chemotherapy, and 27 patients (67.5%) had perioperative chemotherapy, respectively. Seventy-nine (94%) out of eighty-four patients underwent gastrectomy: fourty-one (51.9%) patients in the group with incomplete adhesion and thirty-eight (48.1%) patients in the group with total compliance (Figure 2).

### 3.3. Relationship between Patient/Cancer/Chemotherapy and Compliance

Table S1 reports a univariable analysis that considers different potential factors which could affect treatment interruption. Patients with basal SII ≥ 490 (*p* = 0.017), basal PLR ≥ 152 (*p* = 0.017), the second value of PLR ≥ 131 (*p* = 0.007), the second value of dNLR ≥ 1.75 (*p* = 0.030) were more frequently associated with an interruption of chemotherapy. Changes tending to increase SII (*p* = 0.032), PLR (*p* = 0.034), and LMR (*p* = 0.025), changes (increasing or decreasing) of dNLR (*p* = 0.045) compared to the cut-off were associated with an interruption of chemotherapy, as well as patients who underwent a CF or FOLFOX4/6 regimen (*p* = 0.016), while patients with increased monocytes between the first and second cycles appeared less likely to interrupt chemotherapy (*p* = 0.006). Table 1 reports univariable and multivariable models considering the risk of interruption as a dependent variable; some consideration was made about collinearity among different factors that were statistically significant in univariable analysis: a multivariable model with both SII and PLR was not performed, because the SII and PLR were associated (*p* < 0.001), because they are calculated taking into account platelets and lymphocytes and were both associated with the risk of treatment interruption as reported in the Supplementary Table S1. LMR was not included in the final model because it was calculated taking account of lymphocytes and monocytes that were considered elsewhere while dNLR variation was excluded due to the collinearity. AIC and BIC criteria show that the model with PLR had a better fit (AIC: 1.298; BIC: −231.692). 

The multivariable analysis showed there was a higher risk of interruption for patients who had higher values of PLR compared to the identified cut-off both at pretreatment and second cycle evaluation (OR: 5.03; 95% CI: 1.34–18.89; *p* = 0.017), as well as for patients who had a lower PLR than the identified cut-off at pretreatment evaluation and had a higher PLR value than the cut-off at second cycles (OR: 4.64; 95% CI: 1.02–21.02; *p* = 0.047).

### 3.4. Relationship between Patient/Cancer Characteristics/Chemotherapy and Reasons of Non-Adherence

A further descriptive analysis (Table S2) was made to show the relationships among patient characteristics age, sex, PS, blood values and flogistic indicators, cancer characteristics (site of disease on stomach, grading of cancer, cTNM), type of chemotherapy and compliance. The incomplete-compliance patients were divided into four subgroups based on the cause of non-adherence to treatment (due to toxicity, the patient’s decision, the clinician’s decision or miscellaneous). The data were descriptive only, due to the small number of patients in each group. In the same way, it was considered a variation of these values and there was no significant correlation with compliance. 

### 3.5. Chemotherapies Toxicity and Surgical Complications as Cause of Discontinuation

The most common non-surgical adverse events grade (G)1–G4 reported by patients with at least one cycle of treatment and drop out are shown in Table 2. 

Out of 79 operated GC patients, surgical complications resulted in 24 patients (30.3%): 11 patients (25%) were in the incomplete compliance group and 13 patients (32.5%) were in the total compliance group. If we consider all 24 patients with surgical complications, 16 patients (20.2%) and 8 patients (10.1%) had undergone perioperative and neoadjuvant chemotherapy, respectively. Surgical complications were associated with the suspension of post-surgical treatment in 4 out of 16 patients in the perioperative group. None of the patients died within 30 days of surgery. Four patients (4.7%) out of eighty-four were not operated on because of disease progression and a fifth patient refused the gastrectomy despite the disease being in response to a CT scan. 

### 3.6. Relationship between Pathological Regression, Sarcopenia and Compliance

Considering the SMI evaluated before the first cycle of chemotherapy (pre-SMI) and before surgery (post-SMI), sarcopenia was present in 38 patients (50.7%), while it was present in 47 (60%) out of 75 evaluable patients at the CT scan. A reduction was observed for SMI, VATI and SATI in the overall case series among the first and second CT scan evaluations, while a slight increase was shown for IMATI (Table 3).

Becker regression was neither affected by a decrease in SMI ≥ 5% (*p* = 0.867) nor by a decrease of adipose-tissue indicators ≥ 5% (VATI *p* = 0.762; SATI *p* = 0.144; IMATI *p* = 0.388).

Table 4 reports an analysis between groups of treatment adherence and Becker regression. Total compliance with chemotherapy was neither affected by a decrease in SMI ≥ 5% (*p* = 0.426) nor by a decrease in adipose-tissue indicators ≥ 5% (VATI *p* = 0.292; SATI *p* = 0.426; IMATI *p* = 1.000). 

### 3.7. Assessment of Potential Bias

An analysis was conducted to assess if the risk of interruption was different for patients enrolled inside or outside the GASTRODOC trial through a univariable logistic model. We found that patients treated outside the clinical study had a higher risk of interruption (OR: 2.27; 95% CI: 0.88–5.84), but this data was not statistically significant (*p* = 0.090). 

Similarly, we verified whether patients treated with perioperative chemotherapy had a higher risk than those receiving neoadjuvant chemotherapy, but the risk of discontinuing treatment was not significant (*p* = 0.060) between the two groups.

### 3.8. Time to Event Outcome

**Figure 3:**
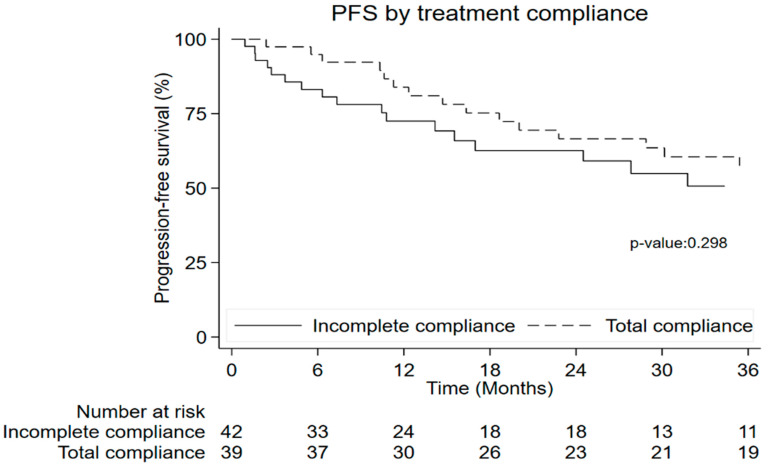
Progression-free survival by treatment compliance.

Eighty-one patients were evaluated for PFS, and thirty-five patients had a progression event confirmed. Treatment adherence was not associated with PFS (*p* = 0.298) (Figure 3). 

**Figure 4:**
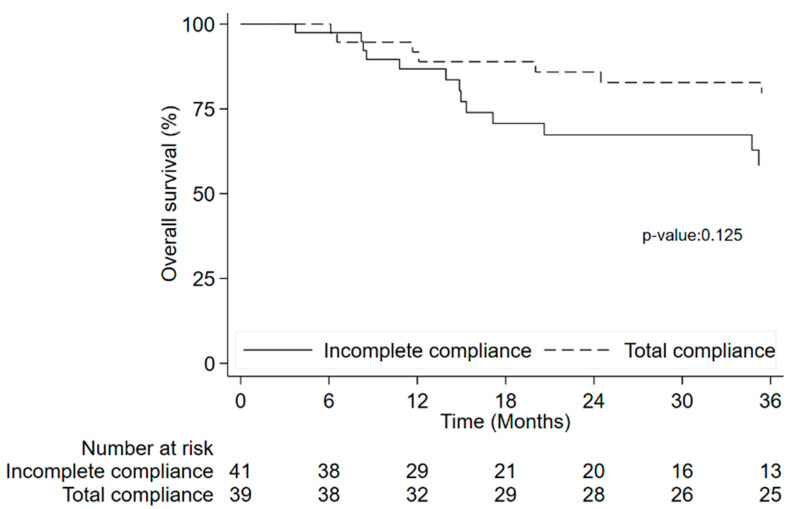
Overall survival by treatment compliance.

Eighty patients were evaluable for OS analysis and twenty-six patients died. Treatment adherence was not associated with OS (*p* = 0.125) (Figure 4). 

Patients with incomplete compliance is a heterogeneous group (patient decision, clinical decision, toxicity, miscellaneous). The comparison in terms of time to event outcomes between total compliance and each distinct reason of incomplete compliance showed a difference between all groups (*p* = 0.045 for PFS and *p* = 0.034 for OS). HR for each subgroup calculated in further analysis showed an higher HR both for PFS and OS in the miscellaneous subgroup than to the patients who completed the treatment [HR 2.83 (95% CI: 1.28–6.29), *p* = 0.010 for PFS and HR 3.51 (95% CI: 1.42–8.63), *p* = 0.006 for OS], while the other subgroups didn’t shown a difference respect to compliant patients group.

Patients with pretreatment sarcopenia had a median PFS equal to 30.2 months (95% CI: 14.1-not estimable), while patients without pretreatment sarcopenia at the basal time had a median PFS equal to 89.3 months (95% CI: 22.0-not estimable) (*p* = 0.228). We also analyzed the baseline values and variations of SMI, but PFS was similar between patients who increased SMI or reduced by ≤5% (median PFS was not reached) and patients who decreased by >5% (median PFS 36.4 months; 95% CI: 18.7-not estimable; *p* = 0.410). 

Patients with pretreatment sarcopenia had a median OS equal to 89.3 months (95% CI: 60.5-not estimable), while median OS for patients without sarcopenia was not yet reached. OS was similar between patients who increased SMI or reduced ≤5% (median OS was not reached) and patients who decreased >5% (median OS 88.2 months; 95% CI: 40.8-not estimable).

## 4. Discussion

The elements that could affect adherence to therapy may depend mainly on individual factors, toxicity due to chemotherapy or surgical complications. The results of this study show that, among the biological indicators, changes in the value of PLR with a tendency towards increasing compared to the cut-off appear to be an immediate indicator of incomplete compliance with neoadjuvant/perioperative treatment. 

The GASTRODOC tried to tackle the problem of compliance with treatment [14] to see if the neoadjuvant approach was better than the perioperative one. The GASTRODOC phase II randomized trial showed that a neoadjuvant approach with four cycles of chemotherapy was more frequently completed and more active than the perioperative approach, but there were still high rates of program interruption in both the neoadjuvant arm (28.3%) and even more so in the perioperative arm (46.7%). If we consider all the patients that interrupted the GASTRODOC study, there were 34 patients (37.4%), while in this study there were 44 patients (52.4%). These data confirm the need to investigate the causes of suspension further. 

If we compare the GASTRODOC to this study, we can see that the rate of dropping out due to patient decision was 5.5% vs. 9.5%; for clinician decisions, 7.7% vs. 8.3%; for toxicity, 11% vs. 16.7%; for miscellaneous, 13.2% vs. 17.9%, respectively. 

We were looking for easily recoverable indicators in clinical practice, which could be useful for predicting compliance with treatment. Consequently, we considered blood tests that were normally correlated with a good state of health and the ability to withstand the treatment. In the same way, we considered CT scans to assess sarcopenia and the loss of adipose tissue. 

To our knowledge, there are no studies that correlate blood ratios with the ability to adhere to neoadjuvant/perioperative chemotherapy, while a growing number of studies have shown that systemic inflammatory response markers, including high NLR, high PLR, elevated dNLR [26], high SIRI [27], low SII [28] and low LMR [29] play an important role in tumor development and prognosis in GC. 

In this study, PLR was a significant marker for compliance with treatment in univariable and multivariate analysis. Our study illustrates the correlation between high PLR and incomplete compliance to treatment starting from the consideration that a high PLR reflects a decrease in the number of lymphocytes and/or an elevated number of platelets. Platelets might stimulate tumor generation and promote metastasis through creating angiogenic factors, for example, platelet-derived growth factor (PDGF) and vascular endothelial growth factor (VEGF) [30]. Lymphocytes have an important role in cancer immune surveillance and in preventing the development of malignancy [31]. An increased platelet count reflects inflammation and high thrombophilic diathesis, while lymphocytopenia is associated with malnutrition and cellular immunosuppression [32]. Malnutrition is a major cause of delayed wound healing [33]. We think that these observations could explain tumor growth and subsequent progression of the disease, gastric perforations, surgical complications, thromboembolism and loss of weight. For that, we grouped them among the various causes of dropout. 

Previously, we tried to explain the miscellaneous cases of treatment discontinuation; now, we will focus on patient decision-making and clinical decision-making. If we consider the patient’s decision in the FLOT trial [4], there were seventeen patients (12.4%) in the ECF/ECX group and twenty patients (15.6%) in the FLOT group, while the investigator’s decision was the cause of suspension of chemotherapy in 4.3% and 2.3% in the ECF/ECX and FLOT, respectively. In our study, the patient’s decision to suspend chemotherapy resulted in eight patients (9.5%), while the clinician’s decision was in seven patients (8.3%). The high percentage of patient decisions leads us to think that there is a need for a psychological basal evaluation and support during treatment. A trial by Allen proposed a prehabilitation program comprising supervised physical exercises and psychological coaching. More prehabilitation patients completed neoadjuvant therapy compared to those who were not prehabilitated [34].

The high percentage of clinical decisions leads us to think that, although the therapy schedules are manageable and are now known, there is concern that the patient cannot be brought to an adequate operable condition. Clinicians may also decide to stop chemotherapy because of suspected disease progression at an intermediate CT scan. Therefore, they are studying GC maximum tumor diameter reduction at CT examinations during neoadjuvant treatment [35].

Several indicators such as Hgb, TBIL and eGFR were found to be prognostic factors in GC, whereas there is no information about its ability to adhere to treatment. 

Studies reported that preoperative anemia was negatively correlated with quality of life and prognosis in patients with cancer [36]. In our study, Hgb < 11 g/dL and its dynamic change did not correlate with either compliance or objective response, and it seems to confirm the unclear significance of preoperative anemia in GC.

Low eGFR may reflect the compromised function of major organs, which is associated with host vulnerability [22]. In a retrospective study, eGFR < 63.2 mL/min/1.73 m^2^ was an independent risk factor for postoperative complications in multivariable analysis (odds ratio 4.67; 95% confidence interval 2.16–10.5; *p* < 0.001) in GC patients [37]. In our experience, the median age was 67.2 years (22.8–80.3), similar to the experience of Tanaka et al. [37], but eGFR and its dynamic change did not correlate with compliance.

Serum bilirubin is an end product of heme metabolism. Because of its anti-inflammation, antioxidant and antiproliferative effects, bilirubin acts as a protective factor against carcinogenesis [38]. In our experience, TBIL did not add compliance information. 

After considering the blood values, we subsequently investigated the patient’s nutritional status. According to the revised European consensus [39] on the definition and diagnosis of sarcopenia recommended by EWGSOP2, sarcopenia was defined by low muscle strength plus low muscle quantity or quality. Muscle quality is a relatively new term, referring to changes in muscle architecture and composition [40].

In our study, there were 38 patients (50.7%) with sarcopenia based on SMI only. Several studies have reported a high prevalence of sarcopenia at diagnosis in patients with gastrointestinal cancers [41,42,43]. Comparison of sarcopenia prevalence among studies is rather difficult because of the use of different methodologies: axial CT cross-sectional imaging of TMA, dual-energy X-ray absorptiometry or a combination of anthropometric and physical performance measurements [44,45]. Furthermore, even when the same methodology is used, different cut-offs for sarcopenia are often used. In the literature, the prevalence of sarcopenia in GC patients ranges from 12.5% to 69.8% at diagnosis. Additionally, the prevalence of sarcopenia increased following neoadjuvant chemotherapy. Awad et al. [46] reported an increase in sarcopenia prevalence from 57% pre-chemotherapy to 79% in patients with esophagogastric cancer. In our experience, sarcopenia based on SMI with a CT scan before surgery (post-SMI) increased to 60%. 

Several studies have shown a relationship between skeletal muscle mass depletion and treatment toxicity [47,48]. The mechanisms through which chemotherapy may impact body composition remain poorly understood. A possible explanation is that the acceleration of muscle mass loss could be associated with the production of proinflammatory cytokines including IL-6, IL-10 and TNF-alpha [49]. Just as blood values are essential to tolerate chemotherapy, sarcopenia is a significant predictor of dose-limiting toxicity [50]. Dose-limiting toxicity is defined as intolerable toxicity, requiring the postponement of treatment, a drug dose reduction or a definitive interruption of drug administration. In our experience, SMI does not correlate with adhesion to treatment probably due to compliance being considered differently (number of cycles planned/administered vs. dose-limiting toxicity).

We looked for a correlation between indicators of fat loss (SATI, VATI, IMATI) or sarcopenia (SMI) and incomplete compliance but we did not find it. Perhaps a greater standardization of the definition of sarcopenia and adipose tissue depletion was also needed to perform the study.

The next point to consider is the type of chemotherapy as a negative prognostic factor for compliance. In this experience, FOLFOX/CF was associated with incomplete adhesion at univariable analysis, even if it appears curious. In the FLOT trial [4], the most common non-surgical G3–4 adverse event was neutropenia (38%) in the ECF/ECX group vs. 52% in the FLOT group, and hospitalization for toxicity occurred in 94 patients (26%) in the ECF/ECX group and 89 patients (25%) in the FLOT group. In a phase II study with FOLFOX6 as neoadjuvant chemotherapy for local GC, neutropenia G3–4 was present in 6.9% of patients [51], but this trial was set with a maximum number of three cycles. In the current study, neutropenia G3–4 was present in 31.9% of patients, but we considered this to be a heterogeneous group for chemotherapy schedules. It could be possible that FOLFOX is a negative factor for compliance because in clinical practice, we usually propose this treatment to elderly patients and/or patients that the clinician feels are fragile.

Differently from other studies where low NLR [52] or low PLR and high LWR [53] predicted a high objective response rate and pathologic response rate, in our study, the pathological response did not correlate with either inflammatory indices or anemia, TBIL or eGFR. The different conclusions regarding pathological response might depend on the different kinds of chemotherapies and the different cut-off values of inflammatory indices. One study [54] reported that was no significant association of body composition changes (BMI, SMI and VATI) with treatment outcomes concerning treatment delays, RECIST response, toxicity and the completion of perioperative FLOT treatment, therefore confirming the findings of similar studies [46,55]. On the contrary, Rinninella et al. found a worse pathological response was significantly associated with an SMI decrease (*p* = 0.01) in a few patients [54]. In our study, Becker regression was not modified by the worsening of sarcopenia and depletion of adipose tissues ≥5%. An interesting point of our study was that incomplete compliance the chemotherapy did not seem to cause a worse pathological response, and this seems to reinforce the fact that neoadjuvant chemotherapy is the most important part of the treatment.

If we considered the comparison in term of time to event outcomes between total compliance and each of the four reasons of incomplete compliance, there would be a difference between the groups (*p* = 0.045 for PFS and *p* = 0.034 for OS), and the miscellaneous group showed a high risk of poor survival. However, considering that the other reasons of non-adhesion have a low number of patients, we believe that it is useful to study a bigger sample to analyze the relationship.

This study has some limitations. First of all, it was a single-center and retrospective study, and related to this, a relatively small number of cases were considered. In addition, we considered different kinds of chemotherapy and treatments (neoadjuvant and perioperative). We did not consider a psychological evaluation and/or survey of quality of life and the correct algorithm to make a diagnosis of sarcopenia. A more detailed observation could have come from dihydropyrimidine dehydrogenase, which was only available for 26 patients (31%) because its evaluation became daily practice only at a later stage. Additionally, we did not report comorbidities. Despite these limitations, this is the second study after Rinninella et al. [54], but it is the first in terms of the number of patients and the first to evaluate body composition changes and their impact on short-term outcomes in locally advanced GC patients who have undergone preoperative chemotherapy. Another interesting point of our study is that first, we pay attention to common pre-treatment indicators as prognostic markers for compliance with the treatment. We are conducting the European project Grammy (InteGRAtive analysis of tuMor, Microenvironment, immunity), funded by ERA PerMEd JTC2019, for evaluating personalized response prediction for neoadjuvant GC patients. We also consider their psychological status and quality of life.

## 5. Conclusions

In conclusion, patients dropping out of neoadjuvant/perioperative treatment has been frequently observed in GC. Despite the limitations of our analysis, changes in the value of PLR, which tends to increase compared to the cut-off, appear to be an immediate indicator of incomplete compliance with neoadjuvant/perioperative treatment, while sarcopenia and the depletion of adipose tissue seem not to be associated with compliance with chemotherapy. Further studies are needed to focus on the indicators of compliance to better understand what leads patients or clinicians to suspend treatment. Ideally, these patients should maintain contact with their oncologist or surgeon to receive greater support. It is also necessary to define the standard cut-off of inflammatory response markers and prospectively consider a nomogram that can predict adhesion to treatment and which can also be associated with pathological response indicators.

## Figures and Tables

**Table 1:** Univariable and multivariable models for risk of interruption.

Covariates	N° of Cases (%)	Univariable Models		Multivariable Model	
		OR (95% CI)	*p*-Value	OR (95% CI)	*p*-Value
Treatment					
CF/FOLFOX4/6	21 (25.0)	5.10 (1.02–25.5)	0.047	6.15 (0.94–39.97)	0.057
DOC	44 (52.4)	1.00 (0.27–3.77)	1.000	1.27 (0.26–6.19)	0.770
ECX/ECF/EOX	8 (9.5)	0.40 (0.05–2.93)	0.367	0.40 (0.04–3.68)	0.419
FLOT	11 (13.1)	1.00		1.00	
PLR variation variation from baseline to 2nd *					
Both lower than cut off	22 (26.8)	1.00		1.00	
From lower to higher	17 (20.7)	2.99 (0.79–11.42)	0.107	4.64 (1.02–21.02)	0.047
From higher to lower	7 (8.5)	1.99 (0.34–11.70)	0.442	1.18 (0.13–10.52)	0.881
Both higher than cut off	36 (44.0)	5.33 (1.66–17.12)	0.005	5.03 (1.34–18.89)	0.017
Monocyte variation from baseline to 2nd *					
Decrease	24 (29.3)	1.00		1.00	
Increase	58 (70.7)	0.24 (0.08–0.68)	0.008	0.38 (0.09–1.51)	0.171

* PLR variation was calculated comparing PLR to the identified cut-off both at pretreatment (cut-off: 152) and second cycle evaluation (cut-off: 131); monocyte variation was calculated comparing pretreatment and second cycle evaluation monocyte value.

**Table 2:** Targeted adverse events reported by patients with at least one cycle of chemotherapy.

Adverse Event	No Cycle Completed *n* = 44 (%)		All Completed Cycles *n* = 40 (%)	
	G1-G2	G3-G4	G1-G2	G3-G4
Neutropenia	5 (10.2)	15 (30.6)	6 (17.1)	11 (31.4)
Interruption due to toxicity	3	3	-	-
Febrile neutropenia	0 (0.0)	1 (2.0)	0 (0.0)	0 (0.0)
Interruption due to toxicity	0	1	-	-
Leukopenia	1 (2.0)	3 (6.1)	1 (2.9)	0 (0.0)
Interruption due to toxicity	1	1	-	-
Anemia	4 (8.2)	1 (2.0)	2 (5.7)	1 (2.9)
Interruption due to toxicity	1	0	-	-
Thrombocytopenia	2 (4.1)	0 (0.0)	3 (8.6)	0 (0.0)
Interruption due to toxicity	2	0	-	-
Asthenia	13 (26.5)	4 (8.2)	13 (37.1)	4 (11.4)
Interruption due to toxicity	8	1	-	-
Fever	1 (2.0)	0 (0.0)	3 (8.6)	0 (0.0)
Interruption due to toxicity	0	0	-	-
Nausea	13 (26.5)	1 (2.0)	12 (34.3)	2 (5.7)
Interruption due to toxicity	5	0	-	-
Vomiting	6 (12.2)	1 (2.0)	8 (22.9)	1 (2.9)
Interruption due to toxicity	2	0	-	-
Diarrhea	16 (32.7)	4 (8.2)	16 (45.7)	3 (8.6)
Interruption due to toxicity	4	1	-	-
Stomatitis	9 (18.4)	0 (0.0)	7 (20.0)	0 (0.0)
Interruption due to toxicity	2	0	-	-
Rash	2 (4.1)	0 (0.0)	1 (2.9)	0 (0.0)
Interruption due to toxicity	0	0	-	-
Pruritus	1 (2.0)	0 (0.0)	1 (2.9)	0 (0.0)
Interruption due to toxicity	0	0	-	-
Bronchospasm	1 (2.0)	0 (0.0)	0 (0.0)	1 (2.9)
Interruption due to toxicity	0	0	-	-
Edema	3 (6.1)	0 (0.0)	0 (0.0)	0 (0.0)
Interruption due to toxicity	1	0	-	-
Paresthesia	5 (10.2)	0 (0.0)	5 (14.3)	0 (0.0)
Interruption due to toxicity	2	0	-	-
Hand-foot syndrome	3 (6.1)	1 (2.0	4 (11.4)	1 (2.9)
Interruption due to toxicity	1	1	-	-
Weight	1 (2.0)	0 (0.0)	0 (0.0)	0 (0.0)
Interruption due to toxicity	1	0	-	-
Liver	2 (4.1)	0 (0.0)	0 (0.0)	0 (0.0)
Interruption due to toxicity	1	0	-	-
Renal	1 (2.0)	0 (0.0)	0 (0.0)	0 (0.0)
Interruption due to toxicity	1	0	-	-
Pain	5 (10.2)	0 (0.0)	10 (28.6)	0 (0.0)
Interruption due to toxicity	1	0	-	-

**Table 3:** Changes in skeletal muscles indices between baseline and post-therapy.

Variable	No Cycle Completed *n* = 39 (%)	All Completed Cycles *n* = 36 (%)	Overall *n* = 75 (%)	*p*-Value
SMI				
Reduced or equal	31 (79.5)	28 (77.8)	59 (78.7)	0.857
Increased	8 (20.5)	8 (22.2)	16 (21.3)	
VATI				
Reduced or equal	22 (56.4)	27 (75.0)	49 (65.3)	0.091
Increased	17 (43.6)	9 (25.0)	26 (34.7)	
SATI				
Reduced or equal	24 (61.5)	25 (69.4)	49 (65.3)	0.472
Increased	15 (38.5)	11 (30.6)	26 (34.7)	
IMATI				
Reduced or equal	16 (41.0)	14 (38.9)	30 (40.0)	0.850
Increased	23 (59.0)	22 (61.1)	45 (60.0)	

**Table 4:** Analysis between groups of treatment adherence and Becker regression.

Variable	Becker 1 *n* = 19 (%)	Becker 2 *n* = 23 (%)	Becker 3 *n* = 29 (%)	Overall * n = 71 (%)	*p*-Value
Treatment compliance					
Incomplete compliance	8 (42.1)	10 (43.5)	18 (62.1)	36 (50.7)	0.281
Total compliance	11 (57.9)	13 (56.5)	11 (37.9)	35 (49.3)	
Treatment adherence					
All completed cycles	11 (57.9)	13 (56.5)	11 (38.0)	35 (49.2)	0.172
Incomplete for toxicity	4 (21.1)	4 (17.4)	3 (10.3)	11 (15.5)	
Incomplete for patient decision	3 (15.8)	1 (4.4)	2 (6.9)	6 (8.5)	
Incomplete for investigator decision	0 (0.0)	3 (13.0)	3 (10.3)	6 (8.5)	
Incomplete for other reasons (miscellaneous)	1 (5.2)	2 (8.7)	10 (34.5)	13 (18.3)	

* Seventy-one patients out of seventy-nine operated GC patients had a well-defined Becker regression, while for the remaining eight patients, the pathologist did not report the kind of pathological response.

## Data Availability

The datasets generated and/or analyzed during the current study are available from the corresponding author upon reasonable request.

## Supplementary Materials

The following supporting information can be downloaded at: https://www.mdpi.com/article/10.3390/nu15163604/s1, Table S1: Univariate analysis between different indicators and compliance according to completed vs. not completed treatment in terms of planned/done cycles. Table S2: Patient characteristics according to completed treatment (completed cycles vs. interruption by cause).
