# Supplementary material for: Association between Pre-Treatment Biological Indicators and Compliance to Neoadjuvant/Perioperative Chemotherapy in Operable Gastric Cancer
Source: Nutrients. 2023 Aug 17;15(16):3604. doi: 10.3390/nu15163604 (PMC10458231; doi:10.3390/nu15163604)
Supplement: Supplementary file 1 [file nutrients-15-03604-s001.zip › nutrients-2485905-supplementary.pdf]

SUPPLEMENTARY MATERIALS

Table S1. Univariate analysis between different indicators and compliance according to completed vs. not completed treatment in terms of planned/done cycles.

Variable	No cycle completed N=44 (%)	All completed cycles N=40 (%)	Overall N=84 (%)	*p*-value
**Median age in years (range)**	70.0 (22.8-80.2)	65.0 (48.6-75.7)	67.2 (22.8-80.3)	-
**Age at start therapy (years)**				
≤65	15 (34.1)	19 (47.5)	34 (40.5)	0.211
>65	29 (65.9)	21 (52.5)	50 (59.5)	
**Gender**				
Male	25 (56.8)	30 (75.0)	55 (65.5)	0.080
Female	19 (43.2)	10 (25.0)	29 (34.5)	
**Race**				
Caucasian/white	44 (100.0)	39 (97.5)	83 (98.8)	-
Asiatic	0 (0.0)	1 (2.5)	1 (1.2)	
**ECOG (performance status)**				
0	42 (97.7)	34 (89.5)	76 (93.8)	0.181
1	1 (2.3)	4 (10.5)	5 (6.1)	
Unknown	1	2	3	
**Tumor localization**				
Fundus	6 (13.6)	5 (12.5)	11 (13.1)	0.660
Corpus	14 (31.8)	10 (25.0)	24 (28.6)	
Antrum/Pilorus	10 (22.7)	14 (35.0)	24 (28.6)	
Other	14 (31.8)	11 (27.5)	25 (29.8)	
**Tumor differentiation**				

G1	2 (6.1)	1 (3.5)	3 (4.8)	1.000
G2	11 (33.3)	11 (37.9)	22 (35.5)	
G3	18 (54.5)	16 (55.1)	34 (54.8)	
G4	2 (6.1)	1 (3.5)	3 (4.9)	
Unknown	11	11	22	
**cT**				
T2	7 (15.9)	4 (10.0)	11 (13.1)	0.777
T3	26 (59.1)	25 (62.5)	51 (60.7)	
T4	11 (25.0)	11 (27.2)	22 (26.2)	
**cN**				
N0	8 (18.2)	7 (17.5)	15 (17.9)	0.935
N+	36 (81.8)	33 (82.5)	69 (82.1)	
**Lauren histotype**				
Diffuse	7 (25.0)	5 (17.9)	12 (21.4)	0.915
Intestinal	18 (64.3)	20 (71.4)	38 (67.9)	
Mixed	3 (10.7)	3 (10.7)	6 (10.7)	
Unknown	16	12	28	
**Pre NLR**				
NLR<1.60	5 (11.4)	4 (10.0)	9 (10.7)	1.000
NLR≥1.60	39 (88.6)	36 (90.0)	75 (89.3)	
**Before 2nd cycle NLR**				
NLR<3.05	31 (73.8)	35 (87.5)	66 (80.5)	0.118
NLR≥3.05	11 (26.2)	5 (12.5)	16 (19.5)	
Unknown	2	0	2	
**Variation of NLR**				
Both lower than cut off	4 (9.5)	4 (10.0)	8 (9.8)	0.409
From lower to higher	1 (2.4)	0 (0.0)	1 (1.2)	
From higher to lower	27 (64.3)	31 (77.5)	58 (70.7)	
Both higher than cut off	10 (23.8)	5 (12.5)	15 (18.3)	

Unknown	2	0	2	
**Pre MLR**				
MLR <0.5	40 (90.9)	38 (95.0)	78 (92.9)	0.678
MLR ≥0.5	4 (9.1)	2 (5.0)	6 (7.1)	
**Before 2nd cycle MLR**				
MLR <0.44	27 (65.9)	20 (50.0)	47 (58.0)	
MLR ≥0.44	14 (34.1)	20 (50.0)	34 (42.0)	0.148
Unknown	3	0	3	
**Variation of MLR**				
Both lower than cut off	9 (22.0)	6 (15.0)	15 (18.5)	
From lower to higher	3 (7.3)	1 (2.5)	4 (4.9)	
From higher to lower	18 (43.9)	14 (35.0)	32 (39.5)	0.239
Both higher than cut off	11 (26.8)	19 (47.5)	30 (37.1)	
Unknown	3	0	3	
**Pre LMR**				
LMR <2.6	16 (36.4)	9 (22.5)	25 (29.8)	0.165
LMR ≥2.6	28 (63.6)	31 (77.5)	59 (70.2)	
**Before 2nd Cycle LMR**				
LMR <2.25	13 (31.7)	20 (50.0)	33 (40.7)	
LMR ≥2.25	28 (68.3)	20 (50.0)	48 (59.3)	0.094
Unknown	3	0	3	
**Variation of LMR**				
Both lower than cut off	8 (19.5)	9 (22.5)	17 (21.0)	
From lower to higher	7 (17.1)	0 (0.0)	7 (8.6)	
From higher to lower	5 (12.2)	11 (27.5)	16 (19.8)	0.025
Both higher than cut off	21 (51.2)	20 (50.0)	41 (50.6)	
Unknown	3	0	3	
**Pre LWR**				
LWR<0.22	15 (34.1)	9 (22.5)	24 (28.6)	0.240
LWR≥0.22	29 (65.9)	31 (77.5)	60 (71.4)	

**Before 2nd cycle LWR**				
LWR<0.22	11 (26.2)	7 (17.5)	18 (22.0)	
LWR≥0.22	31 (73.8)	33 (82.5)	64 (78.0)	0.342
Unknown	2	0	2	
**Variation of LWR**				
Both lower than cut off	7 (16.7)	4 (10.0)	11 (13.4)	
From lower to higher	7 (16.7)	5 (12.5)	12 (14.6)	0.670
From higher to lower	4 (9.5)	3 (7.5)	7 (8.6)	
Both higher than cut off	24 (57.1)	28 (70.0)	52 (63.4)	
Unknown	2	0	2	
**Pre SII**				
SII<490	10 (22.7)	19 (47.5)	29 (34.5)	0.017
SII≥490	34 (77.3)	21 (52.5)	55 (65.5)	
**Before 2nd cycle SII**				
SII<448	18 (42.9)	25 (62.5)	43 (52.4)	
SII≥448	24 (57.1)	15 (37.5)	39 (47.6)	0.075
Unknown	2	0	2	
**Variation of SII**				
Both lower than cut off	8 (19.1)	13 (32.5)	21 (25.6)	
From lower to higher	2 (4.7)	6 (15.0)	8 (9.8)	
From higher to lower	10 (23.8)	12 (30.0)	22 (26.8)	0.032
Both higher than cut off	22 (52.4)	9 (22.5)	31 (37.8)	
Unknown	2	0	2	
**Pre SIRI**				
SIRI<1.05	18 (40.9)	11 (27.5)	29 (34.5)	0.197
SIRI≥1.05	26 (59.1)	29 (72.5)	55 (65.5)	
**Before 2nd cycle SIRI**				
SIRI<0.78	16 (39.0)	10 (25.0)	26 (32.1)	
SIRI≥0.78	25 (61.0)	30 (75.0)	55 (67.9)	0.176
Unknown	3	0	3	

**Variation of SIRI**				
Both lower than cut off	11 (26.8)	6 (15.0)	17 (21.0)	0.552
From lower to higher	5 (12.2)	5 (12.5)	10 (12.4)	
From higher to lower	5 (12.2)	4 (10.0)	9 (11.1)	
Both higher than cut off	20 (48.8)	25 (62.5)	45 (55.5)	
Unknown	3	0	3	
**Pre dNLR**				
dNLR<1.76	28 (63.6)	23 (57.5)	51 (60.7)	0.565
dNLR≥1.76	16 (36.4)	17 (42.5)	33 (39.3)	
**Before 2nd cycle dNLR**				
dNLR<1.75	30 (70.0)	36 (90.0)	66 (79.5)	0.030
dNLR≥1.75	13 (30.2)	4 (10.0)	17 (20.5)	
Unknown	1	0	1	
**Variation of dNLR**				
Both lower than cut off	23 (53.5)	23 (57.5)	46 (55.4)	0.045
From lower to higher	5 (11.6)	0 (0.0)	5 (6.0)	
From higher to lower	7 (16.3)	13 (32.5)	20 (24.1)	
Both higher than cut off	8 (18.6)	4 (10.0)	12 (14.5)	
**Pre PLR**				
PLR<152.0	15 (34.1)	24 (60.0)	39 (46.4)	0.017
PLR≥152.0	29 (65.9)	16 (40.0)	45 (53.6)	
**Before 2nd cycle PLR**				
PLR<131.0	9 (21.4)	20 (50.0)	29 (35.4)	0.007
PLR≥131.0	33 (78.6)	20 (50.0)	53 (64.6)	
Unknown	2	0	2	
**Variation of PLR**				
Both lower than cut off	6 (14.3)	16 (40.0)	22 (26.8)	0.034
From lower to higher	9 (21.4)	8 (20.0)	17 (20.7)	
From higher to lower	3 (7.1)	4 (10.0)	7 (8.5)	
Both higher than cut off	24 (57.1)	12 (30.0)	36 (44.0)	

**Pre SMI***				
Sarcopenia	20 (51.3)	18 (50.0)	38 (50.7)	0.912
No sarcopenia	19 (48.7)	18 (50.0)	37 (49.3)	
Unknown	5	4	9	
**Post SMI***				
Sarcopenia	22 (56.4)	23 (63.9)	45 (60.0)	0.509
No sarcopenia	17 (43.6)	13 (36.1)	30 (40.0)	
Unknown	5	4	9	
**Pre VATI (cm/m^2)**				
<46	22 (52.4)	18 (47.4)	40 (50.0)	0.654
≥46	20 (47.6)	20 (52.6)	40 (50.0)	
Unknown	2	2	4	
**Post VATI (cm/m^2)**				
<51	19 (47.5)	20 (52.6)	39 (50.0)	0.651
≥51	21 (52.5)	18 (47.4)	39 (50.0)	
Unknown	4	2	6	
**Pre SATI (cm/m^2)**				
<50.8	22 (52.4)	18 (47.4)	40 (50.0)	0.654
≥50.8	20 (47.6)	20 (52.6)	40 (50.0)	
Unknown	2	2	4	
**Post SATI (cm/m^2)**				
<46.0	17 (42.5)	22 (57.9)	39 (50.0)	0.174
≥46.0	23 (57.5)	16 (42.1)	39 (50.0)	
Unknown	4	2	6	
**Pre IMATI (cm/m^2)**				
<3.33	17 (40.5)	23 (60.5)	40 (50.0)	0.073
≥3.33	25 (59.5)	15 (39.5)	40 (50.0)	
Unknown	2	2	4	
**Post IMATI (cm/m^2)**				
<3.40	16 (40.0)	23 (60.5)	39 (50.0)	0.070

≥3.40	24 (60.0)	15 (39.5)	39 (50.0)	
Unknown	4	2	6	
**Treatment**				
CF/ FOLFOX4/6	17 (38.6)	4 (10.0)	21 (25.0)	
DOC	20 (45.4)	24 (60.0)	44 (52.4)	0.016
ECX/ECF/EOX	2 (4.6)	6 (15.0)	8 (9.5)	
FLOT	5 (11.4)	6 (15.0)	11 (13.1)	
**WBC count – pre therapy (cells/mm^3)**				
<4.000	0 (0.0)	1 (2.5)	1 (1.2)	0.476
≥4.000	44 (100.0)	39 (97.5)	83 (98.8)	
**WBC count – 2nd evaluation (cells/mm^3)**				
<4.000	8 (18.6)	6 (15.0)	14 (16.9)	0.661
≥4.000	35 (81.4)	34 (85.0)	69 (83.1)	
Unknown	1	0	1	
**WBC variation from baseline to 2nd**				
**Decrease**	35 (81.4)	28 (70.0)	63 (75.9)	0.305
Increase	8 (18.6)	12 (30.0)	20 (24.1)	
Unknown	1	0	1	
**Lymphocyte count – pre therapy (cells/mm^3)**				
<1.000	2 (4.6)	0 (0.0)	2 (2.4)	0.495
1.000-4.000	42 (95.4)	40 (100.0)	82 (97.6)	
**Lymphocyte count – 2nd evaluation (cells/mm^3)**				
<1.000	3 (7.1)	3 (7.5)	6 (7.3)	
1.000-4.000	39 (92.9)	35 (87.5)	74 (90.3)	0.512
>4.000	0 (0.0)	2 (5.0)	2 (2.4)	
Unknown	2	0	2	
**Lymphocyte variation from baseline to 2nd**				
Decrease	29 (69.1)	22 (55.0)	51 (62.2)	0.255
Increase	13 (30.9)	18 (45.0)	31 (37.8)	
Unknown	2	0	2	

**Monocyte count – pre therapy (cells/mm^3)**				
200-1.000	41 (93.2)	37 (92.5)	78 (92.9)	1.000
>1.000	3 (6.8)	3 (7.5)	6 (7.1)	
**Monocyte count – 2nd evaluation (cells/mm^3)**				
<200	0 (0.0)	1 (2.5)	1 (1.2)	
200-1.000	37 (88.1)	28 (70.0)	65 (79.3)	0.071
>1.000	5 (11.9)	11 (27.5)	16 (19.5)	
Unknown	2	0	2	
**Monocyte variation from baseline to 2nd**				
Decrease	18 (42.9)	6 (15.0)	24 (29.3)	0.006
Increase	24 (57.1)	34 (85.0)	58 (70.7)	
Unknown	2	0	2	
**Platelets – pre therapy (cells/mm^3)**				
<140.000	1 (2.3)	0 (0.0)	1 (1.2)	
140.000-400.000	39 (88.6)	37 (92.5)	76 (90.5)	1.000
>400.000	4 (9.1)	3 (7.5)	7 (8.3)	
**Platelets – 2nd evaluation (cells/mm^3)**				
<140.000	5 (11.6)	1 (2.5)	6 (7.2)	
140.000-400.000	34 (79.1)	36 (92.5)	71 (85.5)	0.201
>400.000	4 (9.3)	2 (5.0)	6 (7.3)	
Unknown	1	0	1	
**Platelet variation from baseline to 2nd**				
Decrease	25 (58.1)	26 (65.0)	51 (61.5)	0.521
Increase	18 (41.9)	14 (35.0)	32 (38.5)	
Unknown	1	0	1	
**Neutrophil count – pre therapy (cells/mm^3)**				
<2.000	0 (0.0)	1 (2.5)	1 (1.2)	
2.000-8.000	42 (95.5)	36 (90.0)	78 (92.9)	0.504
>8.000	2 (4.5)	3 (7.5)	5 (5.9)	
**Neutrophil count - 2nd evaluation (cells/mm^3)**				

<2.000	14 (32.6)	9 (22.5)	23 (27.7)	
2.000-8.000	29 (67.4)	29 (72.5)	58 (69.9)	0.225
>8.000	0 (0.0)	2 (5.0)	2 (2.4)	
Unknown	1	0	1	
**Neutrophil variation from baseline to 2nd**				
Decrease	35 (81.4)	31 (77.5)	66 (79.5)	0.787
Increase	8 (18.6)	9 (22.5)	21 (20.5)	
Unknown	1	0	1	
**Anemia – pre therapy**				
Hgb<11g/dL	16 (36.4)	10 (25.0)	26 (31.0)	0.261
Hgb≥11g/dL	28 (63.6)	30 (75.0)	58 (69.0)	
**Anemia – 2nd evaluation**				
Hgb<11 g/dL	17 (39.5)	13 (32.5)	30 (36.1)	0.505
Hgb≥11 g/dL	26 (60.5)	27 (67.5)	53 (63.9)	
Unknown	1	0	1	
**Hgb variation from baseline to 2nd**				
Decrease	34 (79.1)	33 (82.5)	67 (80.7)	0.784
Increase	9 (20.4)	7 (17.5)	16 (19.3)	
Unknown	1	0	1	
**TBIL – pre therapy (mg/dL)**				
<0.32	18 (40.9)	19 (47.5)	37 (44.1)	0.543
≥0.32	26 (59.1)	21 (52.5)	47 (55.9)	
**TBIL – 2nd evaluation (mg/dL)**				
<0.32	19 (45.2)	18 (46.2)	37 (45.7)	0.934
≥0.32	23 (54.8)	21 (53.8)	44 (54.3)	
Unknown	2	1	3	
**TBIL variation from baseline to 2nd (mg/dL)**				
From <0.32 to <0.32	12 (28.6)	14 (35.9)	26 (32.1)	
From <0.32 to ≥0.32	4 (9.5)	5 (12.8)	9 (11.1)	0.746
From ≥ 0.32 to <0.32	7 (16.7)	4 (10.3)	11 (13.6)	

From ≥0.32 to ≥0.32	19 (45.2)	16 (41.0)	35 (43.2)	
Unknown	2	1	3	
**eGFR –pre therapy (ml/min/1.73 m^2)**				
Higher than 90	12 (27.3)	17 (44.7)	29 (35.4)	
From 60 to 89	28 (63.6)	18 (47.4)	46 (56.1)	0.277
From 30 to 59	4 (9.1)	3 (7.9)	7 (8.5)	
Unknown	0	2	2	
**eGFR –2nd evaluation (ml/min/1.73 m^2)**				
Higher than 90	18 (43.9)	22 (56.4)	40 (50.0)	
From 60 to 89	19 (46.3)	15 (38.5)	34 (42.5)	0.514
From 30 to 59	4 (9.8)	2 (5.1)	6 (7.5)	
Unknown	3	1	4	
**eGFR variation from baseline to 2nd**				
Decrease	12 (29.3)	17 (44.7)	29 (36.7)	0.154
Increase	29 (70.7)	21 (55.3)	50 (63.3)	
Unknown	3	2	5	
**BMI (kg/m^2)**				
<20	3 (6.8)	3 (7.5)	6 (7.2)	0.841
20.0-24.9	15 (34.1)	15 (37.5)	30 (35.7)	
25.0-29.9	20 (45.5)	19 (47.5)	39 (46.4)	
≥30	6 (13.6)	3 (7.5)	9 (10.7)	

BMI; body mass index; HgB; hemoglobin; cN, clinical lymph nodes; cT, clinical tumor; dNLR, neutrophils/white blood cells-neutrophils; ECOG; Eastern Cooperative Oncology Group; eGFR, glomerular filtration rate; IMATI, intermuscular adipose tissue index; LMR, lymphocytes-to-monocytes ratio; LWR, lymphocytes-to-white blood cells ratio; MLR, monocytes-to-lymphocytes ratio; NLR, neutrophils-to-lymphocytes ratio; PLR, platelets-to-lymphocytes ratio; Post, after the last cycle of chemotherapy and then before surgery; Pre, before starting the first cycle of chemotherapy; SATI, subcutaneous adipose tissue index; SII, platelets×neutrophils/lymphocytes; SIRI, neutrophils×monocytes/lymphocytes; SMI, skeletal muscle index; TBIL, total bilirubin; VATI, visceral adipose tissue index; WBC, white blood cells.

[*]Sarcopenia was searched on the definition by Martin *et al.* [21].

Table S2. Patient characteristics according to completed treatment (completed cycles vs. interruption by cause).

Variable	All completed cycles N=40 (%)	Non adherent patients				
		Toxicity N=14 (%)	Patient's decision N=8 (%)	Investigator's decision N=7 (%)	Other N=15 (%)	Overall N=84 (%)
**Median age in years (range)**	65.0 (48.6-75.7)	67.3 (22.8-80.2)	71.1 (49.4-80.2)	72.5 (60.7-79.7)	69.3 (56.1-79.6)	67.2 (22.8-80.3)
**Age at start therapy**						
≤65	19 (47.5)	6 (42.9)	2 (25.0)	2 (28.6)	5 (33.3)	34 (40.5)
>65	21 (52.5)	8 (57.1)	6 (75.0)	5 (71.4)	10 (66.7)	50 (59.5)
**Gender**						
Male	30 (75.0)	10 (71.4)	3 (37.5)	5 (71.4)	7 (46.7)	55 (65.5)
Female	10 (25.0)	4 (28.6)	5 (62.5)	2 (28.6)	8 (53.3)	29 (34.5)
**Race**						
Caucasian/white	39 (97.5)	14 (100.0)	8 (100.0)	7 (100.0)	15 (100.0)	83 (98.8)
Asiatic	1 (2.5)	0 (0.0)	0 (0.0)	0 (0.0)	0 (0.0)	1 (1.2)
**ECOG (performance status)**						
0	34 (89.5)	12 (92.3)	8 (100.0)	7 (100.0)	15 (100.0)	76 (93.8)
1	4 (10.5)	1 (7.7)	0 (0.0)	0 (0.0)	0 (0.0)	5 (6.1)
Unknown	2	1	0	0	0	3
**Tumor localization**						
Fundus	5 (12.5)	3 (21.4)	1 (12.5)	0 (0.0)	2 (13.3)	11 (13.1)
Corpus	10 (25.0)	4 (28.6)	3 (37.5)	4 (57.1)	3 (20.0)	24 (28.6)
Antrum/Pilorus	14 (35.0)	4 (28.6)	2 (25.0)	1 (14.3)	3 (20.0)	24 (28.6)
Other	11 (27.5)	3 (21.4)	2 (25.0)	2 (28.6)	7 (46.7)	25 (29.8)
**Grading**						
G1	1 (3.5)	1 (10.0)	0 (0.0)	1 (25.0)	0 (0.0)	3 (4.8)
G2	11 (37.9)	3 (30.0)	2 (33.3)	2 (50.0)	4 (30.8)	22 (35.5)
G3	16 (55.1)	6 (60.0)	4 (66.7)	1 (25.0)	7 (53.8)	34 (54.8)

G4	1 (3.5)	0 (0.0)	0 (0.0)	0 (0.0)	2 (15.4)	3 (4.9)
Unknown	11	4	2	3	2	22
**cT**						
T2	4 (10.0)	4 (28.6)	0 (0.0)	2 (28.6)	1 (6.7)	11 (13.1)
T3	25 (62.5)	7 (50.0)	6 (75.0)	3 (42.8)	10 (66.7)	51 (60.7)
T4	11 (27.2)	3 (21.4)	2 (25.0)	2 (28.6)	4 (26.6)	22 (26.2)
**cN**						
N0	7 (17.5)	3 (21.4)	2 (25.0)	1 (14.3)	2 (13.3)	15 (17.9)
N+	33 (82.5)	11 (78.6)	6 (75.0)	6 (85.7)	13 (86.7)	69 (82.1)
**Lauren histotype**						
Diffuse	5 (17.9)	2 (28.6)	1 (16.7)	1 (25.0)	3 (27.3)	12 (21.4)
Intestinal	20 (71.4)	3 (42.8)	5 (83.3)	3 (75.0)	7 (63.6)	38 (67.9)
Mixed	3 (10.7)	2 (28.6)	0 (0.0)	0 (0.0)	1 (9.1)	6 (10.7)
Unknown	12	7	2	3	4	28
**Pre NLR**						
NLR<1.60	4 (10.0)	3 (21.4)	0 (0.0)	1 (14.3)	1 (6.7)	9 (10.7)
NLR≥1.60	36 (90.0)	11 (78.6)	8 (100.0)	6 (85.7)	14 (93.3)	75 (89.3)
**Before 2nd cycle NLR**						
NLR <3.05	35 (87.5)	11 (84.6)	3 (42.9)	5 (71.4)	12 (80.0)	66 (80.5)
NLR ≥3.05	5 (12.5)	2 (15.4)	4 (57.1)	2 (28.6)	3 (20.0)	16 (19.5)
Unknown	0	1	1	0	0	2
**Variation of NLR**						
Both lower than cut off	4 (10.0)	2 (15.4)	0 (0.0)	1 (14.3)	1 (6.7)	8 (9.8)
From lower to higher	0 (0.0)	1 (7.7)	0 (0.0)	0 (0.0)	0 (0.0)	1 (1.2)
From higher to lower	31 (77.5)	9 (69.2)	3 (42.9)	4 (57.1)	11 (73.3)	58 (70.7)
Both higher than cut off	5 (12.5)	1 (7.7)	4 (57.1)	2 (28.6)	3 (20.0)	15 (18.3)
Unknown	0	1	1	0	0	2
**Pre MLR**						
MLR <0.50	38 (95.0)	13 (92.9)	8 (100.0)	6 (85.7)	13 (86.7)	78 (92.9)
MLR ≥0.50	2 (5.0)	1 (7.1)	0 (0.0)	1 (14.3)	2 (13.3)	6 (7.1)

**Before 2nd cycle MLR**						
MLR <0.44	20 (50.0)	8 (61.5)	6 (85.7)	4	9	47 (58.0)
MLR ≥0.44	20 (50.0)	5 (38.5)	1 (14.3)	3	5	34 (42.0)
Unknown	0	1	1	0	1	3
**Variation of MLR**						
Both lower than cut off	6 (15.0)	3 (23.1)	2 (28.6)	2 (28.6)	2 (14.3)	15 (18.5)
From lower to higher	1 (2.5)	2 (15.4)	0 (0.0)	0 (0.0)	1 (7.1)	4 (4.9)
From higher to lower	14 (35.0)	5 (38.4)	4 (57.1)	2 (28.6)	7 (50.0)	32 (39.5)
Both higher than cut off	19 (47.5)	3 (23.1)	1 (14.3)	3 (42.8)	4 (28.6)	30 (37.1)
Unknown	0	1	1	0	1	3
**Pre LMR**						
LMR <2.6	9 (22.5)	2 (14.3)	4 (50.0)	4 (57.1)	6 (40.0)	25 (29.8)
LMR ≥2.6	31 (77.5)	12 (85.7)	4 (50.0)	3 (42.9)	9 (60.0)	59 (70.2)
**Before 2nd Cycle LMR**						
LMR <2.25	20 (50.0)	4 (30.8)	1 (14.3)	3 (42.9)	5 (35.7)	33 (40.7)
LMR ≥2.25	20 (50.0)	9 (69.2)	6 (85.7)	4 (57.1)	9 (64.3)	48 (59.3)
Unknown	0	1	1	0	1	3
**Variation of LMR**						
Both lower than cut off	9 (22.5)	1 (7.7)	1 (14.3)	3 (42.9)	3 (21.4)	17 (21.0)
From lower to higher	0 (0.0)	1 (7.7)	2 (28.6)	1 (14.2)	3 (21.4)	7 (8.6)
From higher to lower	11 (27.5)	3 (23.1)	0 (0.0)	0 (0.0)	2 (14.3)	16 (19.8)
Both higher than cut off	20 (50.0)	8 (61.5)	4 (57.1)	3 (42.9)	6 (42.9)	41 (50.6)
Unknown	0	1	1	0	1	3
**Pre LWR**						
LWR <0.22	9 (22.5)	2 (14.3)	3 (37.5)	3 (42.9)	7 (46.7)	24 (28.6)
LWR ≥0.22	31 (77.5)	12 (85.7)	5 (62.5)	4 (57.1)	8 (53.3)	60 (71.4)
**Before 2nd cycle LWR**						
LWR <0.22	7 (17.5)	2 (15.4)	4 (57.1)	2 (28.6)	3 (20.0)	18 (22.0)
LWR ≥0.22	33 (82.5)	11 (84.6)	3 (42.9)	5 (71.4)	12 (80.0)	64 (78.0)
Unknown	0	1	1	0	0	2

**Variation of LWR**						
Both lower than cut off	4 (10.0)	1 (7.7)	2 (28.6)	2 (28.6)	2 (13.3)	11 (13.4)
From lower to higher	5 (12.5)	1 (7.7)	0 (0.0)	1 (14.3)	5 (33.3)	12 (14.6)
From higher to lower	3 (7.5)	1 (7.7)	2 (28.6)	0 (0.0)	1 (6.7)	7 (8.6)
Both higher than cut off	28 (70.0)	10 (76.9)	3 (42.8)	4 (57.1)	7 (46.7)	52 (63.4)
Unknown	0	1	1	0	0	2
**Pre SII**						
SII <490	19 (47.5)	5 (35.7)	1 (12.5)	1 (14.3)	3 (20.0)	29 (34.5)
SII ≥490	21 (52.5)	9 (64.3)	7 (87.5)	6 (85.7)	12 (80.0)	55 (65.5)
**Before 2nd cycle SII**						
SII <448	25 (62.5)	8 (61.5)	1 (14.3)	2 (28.6)	7 (46.7)	43 (52.4)
SII ≥448	15 (37.5)	5 (38.5)	6 (85.7)	5 (71.4)	8 (53.3)	39 (47.6)
Unknown	0	1	1	0	0	2
**Variation of SII**						
Both lower than cut off	13 (32.5)	3 (23.0)	1 (14.3)	1 (14.3)	3 (20.0)	21 (25.6)
From lower to higher	6 (15.0)	2 (15.4)	0 (0.0)	0 (0.0)	0 (0.0)	8 (9.8)
From higher to lower	12 (30.0)	5 (38.6)	0 (0.0)	1 (14.3)	4 (26.7)	22 (26.8)
Both higher than cut off	9 (22.5)	3 (23.0)	6 (85.7)	5 (71.4)	8 (53.3)	31 (37.8)
Unknown	0	1	1	0	0	2
**Pre SIRI**						
SIRI <1.05	11 (27.5)	7 (50.0)	3 (37.5)	2 (28.6)	6 (40.0)	29 (34.5)
SIRI ≥1.05	29 (72.5)	7 (50.0)	5 (62.5)	5 (71.4)	9 (60.0)	55 (65.5)
**Before 2nd cycle SIRI**						
SIRI <0.78	10 (25.0)	6 (45.2)	2 (28.6)	2 (28.6)	6 (42.9)	26 (32.1)
SIRI ≥0.78	30 (75.0)	7 (53.8)	5 (71.4)	5 (71.4)	8 (57.1)	55 (67.9)
Unknown	0	1	1	0	1	3
**Variation of SIRI**						
Both lower than cut off	6 (15.0)	4 (30.8)	2 (28.6)	2 (28.6)	3 (21.4)	17 (21.0)
From lower to higher	5 (12.5)	2 (15.4)	1 (14.3)	0 (0.0)	2 (14.3)	10 (12.4)
From higher to lower	4 (10.0)	2 (15.4)	0 (0.0)	0 (0.0)	3 (21.4)	9 (11.1)

Both higher than cut off	25 (62.5)	5 (38.4)	4 (57.1)	5 (71.4)	6 (42.9)	45 (55.5)
Unknown	0	1	1	0	1	3
**Pre dNLR**						
dNLR <1.76	23 (57.5)	10 (71.4)	4 (50.0)	4 (57.1)	10 (66.7)	51 (60.7)
dNLR ≥1.76	17 (42.5)	4 (28.6)	4 (50.0)	3 (42.9)	5 (33.3)	33 (39.3)
**Before 2nd cycle dNLR**						
dNLR <1.75	36 (90.0)	12 (85.7)	2 (28.6)	5 (71.4)	11 (73.3)	66 (79.5)
dNLR ≥1.75	4 (10.0)	2 (14.3)	5 (71.4)	2 (28.6)	4 (26.7)	17 (20.5)
Unknown	0	0	1	0	0	1
**Variation of dNLR**						
Both lower than cut off	23 (57.5)	9 (64.3)	2 (28.6)	4 (57.1)	8 (53.4)	46 (55.4)
From lower to higher	0 (0.0)	1 (7.1)	2 (28.6)	0 (0.0)	2 (13.3)	5 (6.0)
From higher to lower	13 (32.5)	3 (21.5)	0 (0.0)	1 (14.3)	3 (20.0)	20 (24.1)
Both higher than cut off	4 (10.0)	1 (7.1)	3 (42.8)	2 (28.6)	2 (13.3)	12 (14.5)
Unknown	0	0	1	0	0	1
**Pre PLR**						
PLR <152.0	24 (60.0)	5 (35.7)	2 (25.0)	3 (42.9)	5 (33.3)	39 (46.4)
PLR ≥152.0	16 (40.0)	9 (64.3)	6 (75.0)	4 (57.1)	10 (66.7)	45 (53.6)
**Before 2nd cycle PLR**						
PLR <131.0	20 (50.0)	3 (23.1)	0 (0.0)	1 (14.3)	5 (33.3)	29 (35.4)
PLR ≥131.0	20 (50.0)	10 (76.9)	7 (100.0)	6 (85.7)	10 (66.7)	53 (64.6)
Unknown	0	1	1	0	0	2
**Variation of PLR**						
Both lower than cut off	16 (40.0)	2 (15.4)	0 (0.0)	1 (14.3)	3 (20.0)	22 (26.8)
From lower to higher	8 (20.0)	3 (23.1)	2 (28.6)	2 (28.6)	2 (13.3)	17 (20.7)
From higher to lower	4 (10.0)	1 (7.6)	0 (0.0)	0 (0.0)	2 (13.3)	7 (8.5)
Both higher than cut off	12 (30.0)	7 (53.9)	5 (71.4)	4 (57.1)	8 (53.4)	36 (44.0)
Unknown	0	1	2	0	0	2
**Pre SMI**						
Sarcopenia	18 (50.0)	4 (33.3)	6 (75.0)	4 (57.1)	8 (57.1)	38 (50.7)

No sarcopenia	18 (50.0)	8 (66.7)	2 (25.0)	3 (42.9)	6 (42.9)	37 (49.3)
Unknown	4	2	0	0	3	9
**Post SMI**						
Sarcopenia	23 (63.9)	3 (25.0)	7 (87.5)	6 (85.7)	6 (50.0)	45 (60.0)
No sarcopenia	13 (36.1)	12 (75.0)	1 (12.5)	1 (14.3)	6 (50.0)	30 (40.0)
Unknown	4	2	0	0	3	9
**Pre VATI (cm/m^2)**						
<46	18 (47.4)	8 (61.5)	5 (62.5)	3 (42.9)	6 (42.9)	40 (50.0)
≥46	20 (52.6)	5 (38.5)	3 (37.5)	4 (57.1)	8 (57.1)	40 (50.0)
Unknown	2	1	0	0	1	4
**Post VATI (cm/m^2)**						
<51	20 (52.6)	6 (50.0)	5 (62.5)	3 (42.9)	5 (38.5)	39 (50.0)
≥51	18 (47.4)	6 (50.0)	3 (37.5)	4 (57.1)	8 (61.5)	39 (50.0)
Unknown	2	2	0	0	2	6
**Pre SATI (cm/m^2)**						
<50.8	18 (47.4)	9 (69.2)	4 (50.0)	5 (71.4)	4 (28.6)	40 (50.0)
≥50.8	20 (52.6)	4 (30.8)	4 (50.0)	2 (28.6)	10 (71.4)	40 (50.0)
Unknown	2	1	0	0	1	4
**Post SATI (cm/m^2)**						
<46.0	22 (57.9)	7 (58.3)	3 (37.5)	3 (42.9)	4 (30.8)	39 (50.0)
≥46.0	16 (42.1)	5 (41.7)	5 (62.5)	4 (57.1)	9 (69.2)	39 (50.0)
Unknown	2	2	0	0	2	6
**Pre IMATI (cm/m^2)**						
<3.33	23 (60.5)	7 (53.9)	2 (25.0)	4 (57.1)	4 (28.6)	40 (50.0)
≥3.33	15 (39.5)	6 (46.1)	6 (75.0)	3 (42.9)	10 (71.4)	40 (50.0)
Unknown	2	1	0	0	1	4
**Post IMATI (cm/m^2)**						
<3.40	23 (60.5)	5 (41.7)	1 (12.5)	5 (71.4)	5 (38.5)	39 (50.0)
≥3.40	15 (39.5)	7 (58.3)	7 (87.5)	2 (28.6)	8 (61.5)	39 (50.0)

Unknown	2	2	0	0	2	6
**Pre SUB IMATI VISC (cm/m^2)**						
<114	19 (50.0)	7 (53.9)	5 (62.5)	4 (57.1)	5 (35.7)	40 (50.0)
≥114	19 (50.0)	6 (46.1)	3 (37.5)	3 (42.9)	9 (64.3)	40 (50.0)
Unknown	2	1	0	0	1	4
**Post SUB IMATI VISC (cm/m^2)**						
<103	21 (55.3)	6 (50.0)	5 (62.5)	3 (42.9)	4 (30.8)	39 (50.0)
≥103	17 (44.7)	6 (50.0)	3 (37.5)	4 (57.1)	9 (69.2)	39 (50.0)
Unknown	2	2	0	0	2	6
**Treatment**						
CF/ FOLFOX4/6	4 (10.0)	3 (21.4)	4 (50.0)	5 (71.4)	5 (33.3)	21 (25.0)
DOC	24 (60.0)	9 (64.4)	3 (37.5)	1 (14.3)	7 (46.7)	44 (52.4)
ECX/ECF/EOX	6 (15.0)	1 (7.1)	0 (0.0)	0 (0.0)	1 (6.7)	8 (9.5)
FLOT	6 (15.0)	1 (7.1)	1 (12.5)	1 (14.3)	2 (13.3)	11 (13.1)
**WBC count – pre therapy (cells/mm^3)**						
<4.000	1 (2.5)	0 (0.0)	0 (0.0)	0 (0.0)	0 (0.0)	1 (1.2)
≥4.000	39 (97.5)	14 (100.0)	8 (100.0)	7 (100.0)	15 (100.0)	83 (98.8)
**WBC count - 2nd evaluation (cells/mm^3)**						
<4.000	6 (15.0)	3 (21.4)	2 (28.6)	0 (0.0)	3 (20.0)	14 (16.8)
≥4.000	34 (85.0)	11 (78.6)	5 (71.4)	7 (100.0)	12 (80.0)	69 (83.2)
Unknown	0	0	1	0	0	1
**WBC variation from baseline to 2nd**						
**Decrease**	28 (70.0)	13 (92.9)	5 (71.4)	5 (71.4)	12 (80.0)	63 (75.9)
Increase	12 (30.0)	1 (7.1)	2 (28.6)	2 (28.6)	3 (20.0)	20 (24.1)
Unknown	0	0	1	0	0	1
**Lymphocyte count - pre therapy (cells/mm^3)**						
<1.000	0 (0.0)	0 (0.0)	1 (12.5)	0 (0.0)	1 (6.7)	2 (2.4)
1.000-4.000	40 (100.0)	14 (100.0)	7 (87.5)	7 (100.0)	14 (93.3)	82 (97.6)
**Lymphocyte count - 2nd evaluation (cells/mm^3)**						
<1.000	3 (7.5)	0 (0.0)	1 (14.3)	0 (0.0)	2 (13.3)	6 (7.3)

1.000-4.000	35 (87.5)	13 (100.0)	6 (87.5)	7 (100.0)	13 (86.7)	74 (90.3)
>4.000	2 (5.0)	0 (0.0)	0 (0.0)	0 (0.0)	0 (0.0)	2 (2.4)
Unknown	0	1	1	0	0	2
**Lymphocyte variation from baseline to 2nd**						
Decrease	22 (55.0)	8 (61.5)	6 (85.7)	6 (85.7)	9 (60.0)	51 (62.2)
Increase	18 (45.0)	5 (38.5)	1 (14.3)	1 (14.3)	6 (40.0)	31 (37.8)
Unknown	0	1	1	0	0	2
**Mono count - pre therapy (cells/mm^3)**						
200-1.000	37 (92.5)	13 (92.9)	8 (100.0)	6 (85.7)	14 (93.3)	78 (92.9)
>1.000	3 (7.5)	1 (7.1)	0 (0.0)	1 (14.3)	1 (6.7)	6 (7.1)
**Mono count - 2nd evaluation (cells/mm^3)**						
<200	1 (2.5)	0 (0.0)	0 (0.0)	0 (0.0)	0 (0.0)	1 (1.2)
200-1.000	28 (70.0)	13 (92.9)	7 (100.0)	6 (85.7)	11 (78.6)	65 (79.3)
>1.000	11 (27.5)	1 (7.1)	0 (0.0)	1 (14.3)	3 (21.4)	16 (19.5)
Unknown	0	0	1	0	1	2
**Monocyte variation from baseline to 2nd**						
Decrease	6 (15.0)	5 (35.7)	3 (42.9)	3 (42.9)	7 (50.0)	24 (29.3)
Increase	34 (85.0)	9 (64.3)	4 (57.1)	4 (57.1)	7 (50.0)	58 (70.7)
Unknown	0	0	1	0	1	2
**Platelets - pre therapy (cells/mm^3)**						
<140.000	0 (0.0)	0 (0.0)	0 (0.0)	1 (14.3)	0 (0.0)	1 (1.2)
140.000-400.000	37 (92.5)	13 (92.9)	8 (100.0)	6 (85.7)	12 (80.0)	76 (90.5)
>400.000	3 (7.5)	1 (7.1)	0 (0.0)	0 (0.0)	3 (20.0)	7 (8.3)
**Platelets -2nd evaluation (cells/mm^3)**						
<140.000	1 (2.5)	2 (14.3)	1 (14.3)	0 (0.0)	2 (13.3)	6 (7.2)
140.000-400.000	37 (92.5)	11 (78.6)	6 (85.7)	5 (71.4)	12 (80.0)	71 (85.6)
>400.000	2 (5.0)	1 (7.1)	0 (0.0)	2 (28.6)	1 (6.7)	6 (7.2)
Unknown	0	0	1	0	0	1
**Platelet variation from baseline to 2nd**						
Decrease	26 (65.0)	7 (50.0)	6 (85.7)	2 (28.6)	10 (66.7)	51 (61.5)

Increase	14 (35.0)	7 (50.0)	1 (14.3)	5 (71.4)	5 (33.3)	32 (38.5)
Unknown	0	0	1	0	0	1
**Neutrophils count - pre therapy (cells/mm^3)**						
<2.000	1 (2.5)	0 (0.0)	0 (0.0)	0 (0.0)	0 (0.0)	1 (1.2)
2.000-8.000	36 (90.0)	13 (92.9)	8 (100.0)	7 (100.0)	14 (93.3)	78 (92.9)
>8.000	3 (7.5)	1 (7.1)	0 (0.0)	0 (0.0)	1 (6.7)	5 (5.9)
**Neutrophils count - 2nd evaluation**						
<2.000	9 (22.5)	6 (42.9)	2 (28.6)	2 (28.6)	4 (26.7)	23 (27.7)
2.000-8.000	29 (72.5)	8 (57.1)	5 (71.4)	5 (71.4)	11 (73.3)	58 (69.9)
>8.000	2 (5.0)	0 (0.0)	0 (0.0)	0 (0.0)	0 (0.0)	2 (2.4)
Unknown	0	0	1	0	0	1
**Neutrophil variation from baseline to 2nd**						
Decrease	31 (77.5)	13 (92.9)	4 (57.1)	5 (71.4)	13 (86.7)	66 (79.5)
Increase	9 (22.5)	1 (7.1)	3 (42.9)	2 (28.6)	2 (13.3)	17 (20.5)
Unknown	0	0	1	0	0	1
**Anemia - pre therapy**						
Hgb <11 g/dL	10 (25.0)	5 (35.7)	4 (50.0)	2 (28.6)	5 (33.3)	26 (30.9)
Hgb ≥11 g/dL	30 (75.0)	9 (64.3)	4 (50.0)	5 (71.4)	10 (66.7)	58 (69.1)
**Anemia – 2nd evaluation**						
Hgb <11 g/dL	13 (32.5)	6 (42.9)	3 (42.9)	3 (42.9)	5 (33.3)	30 (36.1)
Hgb ≥11 g/dL	27 (67.5)	8 (57.1)	4 (57.1)	4 (57.1)	10 (66.7)	53 (63.9)
Unknown	0	0	1	0	0	1
**Hemoglobin variation from baseline to 2nd**						
Decrease	33 (82.5)	12 (85.7)	4 (57.1)	6 (85.7)	12 (80.0)	67 (80.7)
Increase	7 (17.5)	2 (14.3)	3 (42.9)	1 (14.3)	3 (20.0)	16 (19.3)
Unknown	0	0	1	0	0	1
**TBIL - pre therapy**						
<0.32	19 (47.5)	6 (42.9)	3 (37.5)	3 (42.9)	6 (40.0)	37 (44.1)
≥0.32	21 (52.5)	8 (57.1)	5 (62.5)	4 (57.1)	9 (60.0)	47 (55.9)
**TBIL - 2nd evaluation**						

<0.32	18 (46.2)	6 (46.2)	3 (42.9)	4 (57.1)	6 (40.0)	37 (45.7)
≥0.32	21 (53.8)	7 (53.8)	4 (57.1)	3 (42.9)	9 (60.0)	44 (54.3)
Unknown	1	1	1	0	0	3
**TBIL variation from baseline to 2nd**						
From <0.32 to <0.32	14 (35.9)	4 (30.8)	2 (28.6)	2 (28.6)	4 (26.7)	26 (32.1)
From <0.32 to ≥0.32	5 (12.8)	1 (7.7)	0 (0.0)	1 (14.2)	2 (13.3)	9 (11.1)
From ≥ 0.32 to <0.32	4 (10.3)	2 (15.3)	1 (14.3)	2 (28.6)	2 (13.3)	11 (13.6)
From ≥0.32 to ≥0.32	16 (41.0)	6 (46.2)	4 (57.1)	2 (28.6)	7 (46.7)	35 (43.2)
Unknown	1	1	1	0	0	3
**eGFR - pre therapy (ml/min/1.73 m^2)**						
Higher than 90	17 (44.7)	5 (35.7)	2 (25.0)	1 (14.3)	4 (26.7)	29 (35.4)
From 60 to 89	18 (47.4)	8 (57.1)	6 (75.0)	6 (85.7)	8 (53.3)	46 (56.1)
From 30 to 59	3 (7.9)	1 (7.1)	0 (0.0)	0 (0.0)	3 (20.0)	7 (8.5)
Unknown	2	0	0	0	0	2
**eGFR - 2nd evaluation (ml/min/1.73 m^2)**						
Higher than 90	22 (56.4)	6 (46.1)	3 (42.9)	4 (57.1)	5 (35.7)	40 (50.0)
From 60 to 89	15 (38.5)	7 (53.9)	4 (57.1)	2 (28.6)	6 (42.9)	34 (42.5)
From 30 to 59	2 (5.1)	0 (0.0)	0 (0.0)	1 (14.3)	3 (21.4)	6 (7.5)
Unknown	1	1	1	0	1	4
**Egfr variation from baseline to 2nd**						
Decrease	17 (44.7)	4 (30.8)	1 (14.3)	4 (57.1)	3 (21.4)	29 (36.7)
Increase	21 (55.3)	9 (69.2)	6 (85.7)	3 (42.9)	11 (78.6)	50 (63.3)
Unknown	2	1	1	0	1	5
**BMI**						
BMI <20.0	3 (7.5)	1 (7.1)	1 (12.5)	0 (0.0)	1 (6.7)	6 (7.0)
BMI 20.0-24.9	15 (37.5)	7 (50.0)	2 (25.0)	3 (42.9)	3 (20.0)	30 (35.7)
BMI 25.0-29.9	19 (47.5)	5 (35.8)	4 (50.0)	4 (57.1)	7 (46.7)	39 (46.4)
BMI ≥30	3 (7.5)	1 (7.1)	1 (12.5)	0 (0.0)	4 (26.6)	9 (10.7)
